# Rapid target foraging with reach or gaze: The hand looks further ahead than the eye

**DOI:** 10.1371/journal.pcbi.1005504

**Published:** 2017-07-06

**Authors:** Jonathan S. Diamond, Daniel M. Wolpert, J. Randall Flanagan

**Affiliations:** 1 Centre for Neuroscience Studies, Queen's University, Kingston, Ontario, Canada; 2 Department of Engineering, University of Cambridge, Cambridge, United Kingdom; 3 Department of Psychology, Queen’s University, Kingston, Ontario, Canada; Imperial College London, UNITED KINGDOM

## Abstract

Real-world tasks typically consist of a series of target-directed actions and often require choices about which targets to act on and in what order. Such choice behavior can be assessed from an optimal foraging perspective whereby target selection is shaped by a balance between rewards and costs. Here we evaluated such decision-making in a rapid movement foraging task. On a given trial, participants were presented with 15 targets of varying size and value and were instructed to harvest as much reward as possible by either moving a handle to the targets (hand task) or by briefly fixating them (eye task). The short trial duration enabled participants to harvest about half the targets, ensuring that total reward was due to choice behavior. We developed a probabilistic model to predict target-by-target harvesting choices that considered the rewards and movement-related costs (i.e., target distance and size) associated with the current target as well as future targets. In the hand task, in comparison to the eye task, target choice was more strongly influenced by movement-related costs and took into account a greater number of future targets, consistent with the greater costs associated with arm movement. In both tasks, participants exhibited near-optimal behaviour and in a constrained version of the hand task in which choices could only be based on target positions, participants consistently chose among the shortest movement paths. Our results demonstrate that people can rapidly and effectively integrate values and movement-related costs associated with current and future targets when sequentially harvesting targets.

## Author summary

Many natural tasks involve a series of decisions about which target to acquire next, either with our gaze or hand. We examined the factors influencing such decisions using a task in which targets of varying value and size are sequentially acquired by eye or hand movements. By developing a probabilistic model of decision-making behavior we show that eye movement decisions are made in isolation, independent of potential future targets, and are primarily determined by target value. In contrast, hand movement decisions consider multiple future targets and are strongly shaped by movement-related costs. By examining decision-making in sequential actions, our results and model represent a significant advance over previous work that has focused primarily on decisions about single actions.

## Introduction

Studies of reach planning and control have focused on movements towards single targets, with theoretical accounts focusing on the minimization of various movement-related costs [for reviews, see 1, 2–5]. However, real-world tasks often involve choosing targets from among multiple alternatives, and therefore not only involve decisions about *how* to move but also *where* to move. Moreover, such tasks often involve a sequence of actions in which choices are made at each step. Although decision-making related to target selection has been extensively studied in the context of eye movement preparation [6–9] and in more cognitive tasks such as the traveling salesperson problem [10–13], comparatively little work has been done on reaching, in which movement-related costs are likely to play a more critical role [14,15]. A handful of reaching studies have examined how values and costs influence the selection of targets in single movements and fixed sequences of movements. This work has shown that when pointing to target configurations that have different reward and penalty regions, people are able to choose their average pointing location to minimize the loss that accrues through the variability of pointing [e.g. 16], but that when reaching to two consecutive targets in a fixed time period, people fail to invest more time in the movement to the more valuable target [17,18]. In addition, when ‘harvesting’ a sequence of targets by maintaining a hand-controlled cursor on each target for a fixed duration, people learn to optimize reward by predicting the required duration [19]. However, to our knowledge no study has investigated the selection of targets during a sequential reach task.

**Fig 1:**
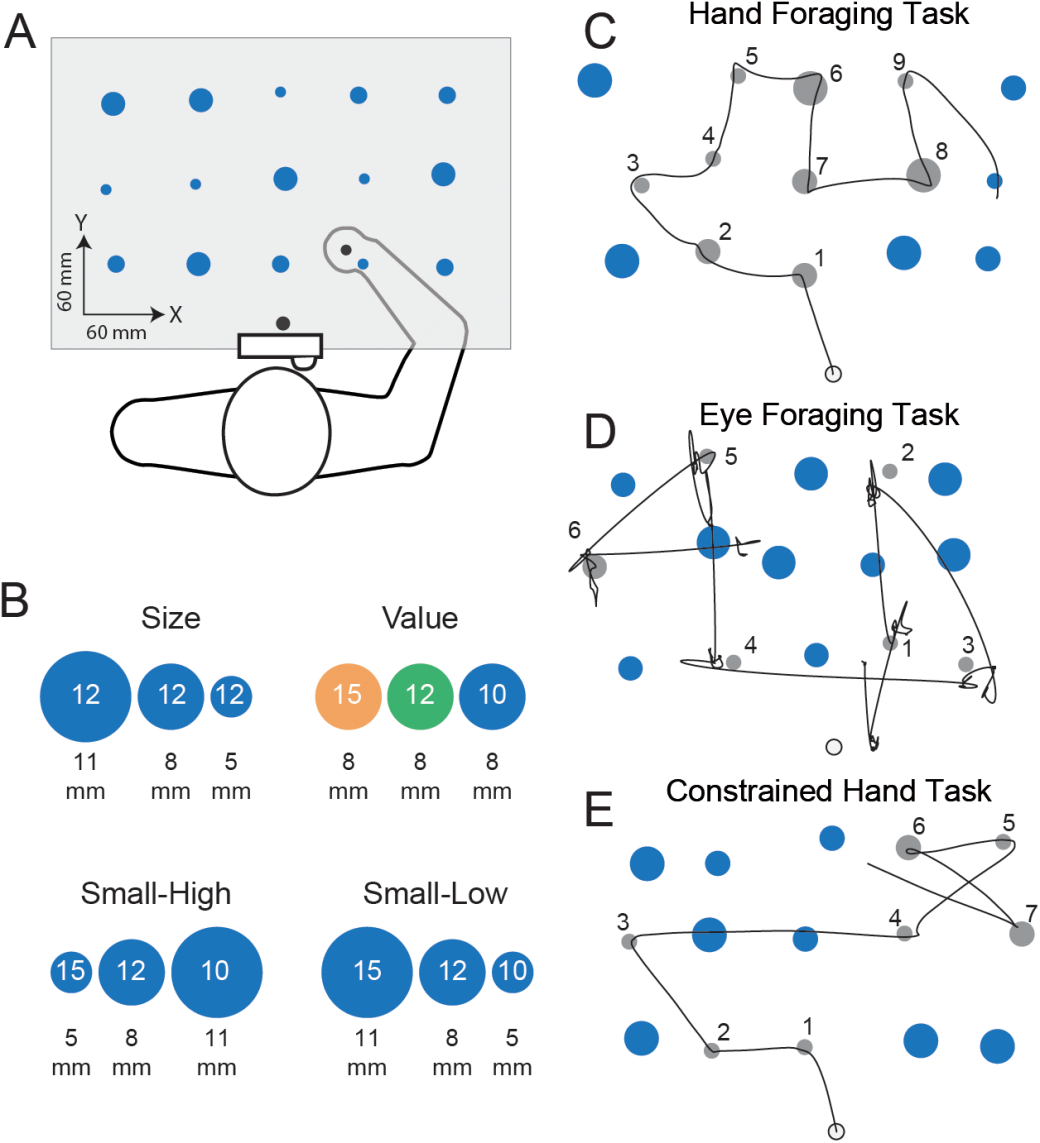
Experimental setup and behavior. **A**, Schematic of the experimental setup and stimuli configuration. In the hand task, participants harvested targets by moving a handle and pressing a button instrumented to the handle to acquire targets. In the eye task, participants harvested targets by fixating a target. **B**, Target size and value pairings featured in the 4 experimental conditions. Five of each target type (size-value pairing) was displayed for a given block and the position of each of the 15 targets was randomized on each trial. **C-E**, Representative traces for the Small-High condition for hand movements or eye movements in each of the three foraging tasks, where grey targets represent those that have been successfully harvested. The numbers indicate the order of harvests.

In performing a task involving the selection of a series of targets, each successive choice decision could be made *de novo* in order to maximize rewards and minimize costs associated with only the next target selection. However, by ‘looking ahead’ and considering the rewards and costs across future potential targets, it may be possible to further optimize performance. Here we assessed sequential decision-making using a movement foraging task in which participants could choose the order in which they harvested from a set of targets of varying size, value and location across the workspace (Fig 1A), either by moving a hand-held handle to a target and clicking a button on the handle (hand task) or making a saccade to a target and fixating it for 150 ms (eye task), with the goal of maximizing reward. Gaze was unconstrained in the hand task. The trial duration was such that, in both tasks, participants could only harvest around half of the targets, placing a premium on efficient decision-making. We examined four conditions: two in which only the size or only the value of targets varied, and two in which both size and value varied in either a correlated or anti-correlated manner (Fig 1B). We evaluated performance using a probabilistic model, inspired by optimal foraging theory [20,21], that predicts target-by-target harvesting probabilities based on rate of reward, costs associated with target distance and size, and decision noise. A key feature of the model is that it can incorporate a number of future successive harvests with temporal discounting; i.e., it can ‘look ahead’. Because moving the hand is more costly, in time and energy, than moving the eyes, we predicted that target choice in the hand task, in comparison to the eye task, would be more strongly influenced by movement-related costs and would take into account a greater number of future targets so as to optimize the route through the targets.

## Results

### Representative trials

Fig 1C–1E shows hand or gaze paths for single trials performed by different participants in each of the three foraging tasks in the ‘small-high’ condition in which target size was inversely related to target value. These trials illustrate several general features of the performance we observed in these tasks. Participants exhibited a strong tendency to move between adjacent targets in the hand task but also tended to make relatively small movements in the eye task. Even in the constrained hand task (Fig 1E, in which the targets had to be harvested in order of decreasing value), participants moved to close-by targets where possible. In the hand task, participants also tended to minimize changes in movement direction between successive harvested targets whereas, in the eye task, sharper changes in direction were often observed. One strategy that participants employed to limit changes in hand movement direction was to harvest targets along a roughly circular route. In all three tasks, participants typically harvested most, if not all, of the high value targets. In the hand task, they often harvested medium or, less frequently, low value targets in between the high value targets. In contrast, in the eye task, there was a stronger tendency to harvest the high value targets prior to lower valued targets. The pattern observed in the hand task suggests that participants were aware that they could typically obtain 8 or 9 targets and could therefore harvest some less valuable targets while ensuring that most, if not all, high value targets were harvested. Harvesting a few less valuable targets en route between high value targets often allowed participants to avoid large amplitude hand movements.

### Target preferences

**Fig 2:**
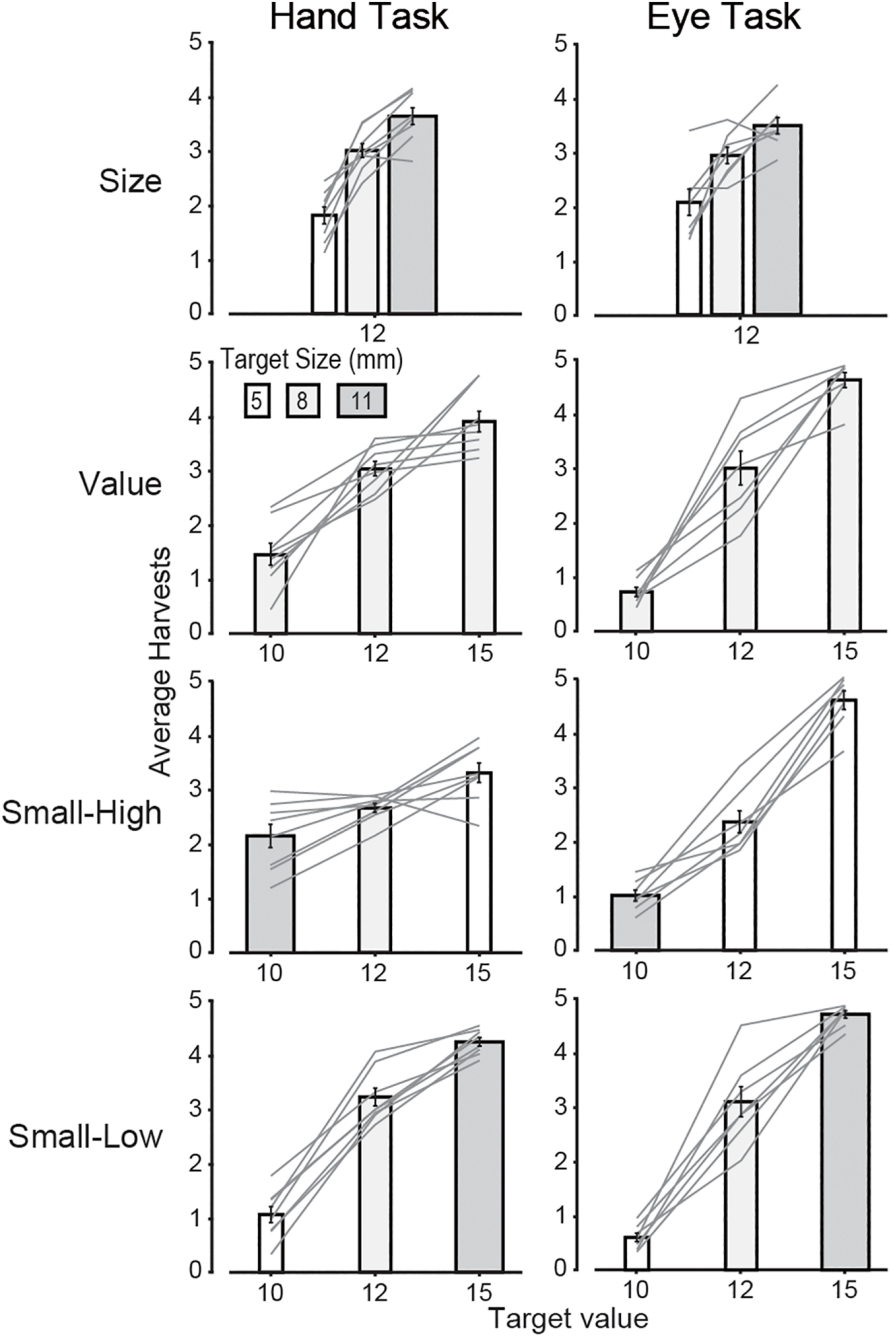
Overall harvesting performance. Average number of harvests per trial for the hand task (left column) and the eye task (right column) for each condition and target type. The bars show the average number of harvests computed from participant means, with error bars representing ±1 SE, and the lines show individual participant means. All bars represent targets of a given size shown for a given value (X-axis), with white, skinny bars representing the smallest target size, light grey, thicker bars representing the medium target size, and thick, dark grey bars representing the largest target size.

Fig 2 shows the mean number of harvests for each condition of the hand and eye foraging tasks. A one-way ANOVA revealed that, overall, the number of targets harvested in the hand (M = 8.4, SE = .16) and eye (M = 8.2, SE = .18) tasks were similar (F_1, 13_ = .12, p = 0.73), allowing us to reasonably compare target preferences across tasks. In both tasks large targets were preferred when only size was varied and high value targets were preferred whenever value was varied (i.e., in the three other conditions). However, in the small-high condition, in which value and size traded off, the preference for high value targets was weaker in the hand task than the eye task. To quantify these results, for each condition we carried out a two-way mixed model ANOVA to assess the effects of target type (3 levels for each condition: see Fig 1B) and task (2 levels: eye or hand) on the number of harvests. In no condition was there a main effect of task (p > 0.05 in all 4 cases). A main effect of target size was found in the size condition (F_2, 26_ = 48.8, p < 0.001) but there was no target by task interaction. A main effect of target value was observed in the value (F_2, 26_ = 104.7, p < 0.001), small-high (F_2, 26_ = 91.6, p < 0.001), and small-low (F_2, 26_ = 280.6, p < 0.001) conditions. A target value by task interaction was found for the value (F_2, 26_ < 5.5, p < 0.05), small-high (F_2, 26_ < 91.6, p < 0.001), and small-low (F_2, 26_ < 4.6, p < 0.05). In these three conditions, participants in the eye task harvested more high value targets than participants in the hand task (Fig 2), possibly because smaller movement-related costs in the eye task allowed participants to harvest targets according to their value, with limited influence of their sizes and/or distances.

Although size similarly influenced hand and gaze target selection in the size condition, when size was paired with value, size had a substantial influence on hand target selection (compare the small-high and small-low conditions) but little influence on gaze target selection. Thus, whereas participants in the eye tasks exhibited a clear preference for large targets when only size varied, this preference was largely superseded by value when value also varied.

### Movement times

For the probabilistic model, we estimated the time required to move to any given target based on its distance and size. Specifically, for each participant, we performed separate regressions between recorded movement time (i.e., time between harvests) and target distance for each target size, where regressions in the arm task were obtained after pooling data from the free choice and constrained tasks. Two-way mixed model ANOVAs were carried out to assess how the slope and intercept varied with task and target size. There was no significant difference (F_1, 13_ = 2.3, p = 0.2) between the slopes in the eye (M = 0.9 s/m; SE = 0.2 s/m) and hand (M = 1.0 s/m; SE = 0.1 s/m) tasks. However, the intercepts were slightly greater (F_1, 13_ = 15.8, p = 0.002) in the eye task (M = 0.3 s; SE = 0.02 s) than the hand task (M = 0.2 s; SE = 0.01 s). Note that with the required fixation duration in the eye task, the time between successive harvests was similar to the hand foraging tasks. The slope (F_2, 26_ = 18.6, p < 0.001) and intercept (F_2, 26_ = 6.0, p = 0.007) also depended on target size. Specifically, the slope and intercept tended to decrease and increase, respectively, as a function of target size.

### Model of harvesting choice

We developed a simple model of harvesting in which the choice of the next harvest target could depend on the distance to each potential target as well as their size and value (which affects the reward rate). We first evaluate the contribution of these factors using the 1-look-ahead version of the model, which only considers the next or immediate harvest when predicting which target will be selected. After establishing, for both the hand and eye tasks, that the full model (with all three of these components) provided a better overall fit than any of the reduced models with one component removed, we then examined versions of the model with 1 to 5 look-ahead steps. That is the next harvest could depend on the weighted combination of these components for up to the next 5 harvests. Such look ahead in the model would allow the eye or hand to forgo short term gains for longer terms gains, for example, by moving to regions of the workspace where there are more rewarding targets. Finally, we evaluated the performance of the best-fit N-look-ahead model by comparing model predictions against actual data.

In all cases, the models were fit to each individual participant from all trials in all four conditions (i.e., size, value, small-high, and small-low) fit together. We only considered up to 8 harvests in each trial because as the number of harvests increased beyond 8, the number of trials decreased sharply. We did not analyze the first harvest of each trial because, in the hand task especially, participants tended to rapidly launch their initial movement in a relatively fixed direction and choose between the one or two targets located in this direction (e.g., the center target in the first row and the target to its left). By limiting decision-making in the initial movement, participants could initiate the task quickly while bringing their hand (or gaze) towards the grid of targets and giving themselves time to select the next target or targets. We used maximum likelihood (MATLAB fminsearch) to fit the model to the entire dataset of 200 trials by 8 harvests (max) for each participant separately.

### One-ahead model

The model assigned, to each available target, a probability that this target would be chosen next. Each selection made by the model began from the most recently harvested target, *i* and considered the sizes, values and positions of the remaining targets, *j* ∈ *H*, where *H* represents the set of remaining non-harvested targets. We defined the distance from target *i* to target *j* as *d*_*ij*_, the value of target *j* as *v*_*j*_ and the size of target *j* as *s*_*j*_. An estimate of the time required to move from target *i* to target *j*, *t*_*ij*_, was derived from linear regressions, relating movement time to movement distance, that were computed separately for each participant and target size. In our sequential target task, we found that the relationship between movement time and distance was close to linear. Rate of reward is known to be a key factor in decision making [e.g. 22, 23] and the rate of reward for selecting target *j* was calculated *as r*_*ij*_ *= v*_*j*_ */ t*_*ij*_. A pure reward based model would only consider rate of reward. In this case, target distance and size can only influence choice via effects on movement duration. However, distance and size may also influence target choice via other costs. For example, distance may be associated with a physical effort cost [24] whereas target size may be associated with a cost linked to planning and controlling more precise movements [25]. To capture this possibility, the cost, *c*_*ij*_, of choosing target *j* as the next harvest was calculated as:

$$C_{j}=c_{ij}=-r_{ij}+w_{1}d_{ij}^{\gamma }-w_{2}s_{j}$$ (1)

That is, the cost depended on the negative reward rate, a penalty associated with distance and a penalty for reaching to smaller targets. The penalty associated with distance captures possible movement-related energy costs. We included the exponent gamma in this term to accommodate the possibility of a nonlinear mapping between effort and distance. A power function was selected because it can capture a wide range of nonlinear functions. We assume there is noise in the decision making process (or calculation of this cost) so that potential harvest targets that have similar costs may be chosen with similar probability. As is commonly used in models of decision making, we used a softmax selection rule [26] so that the probability of choosing target *j* became:

$$P_{j}=\frac{e^{-\beta \cdot C_{j}}}{\sum_{j}e^{-\beta \cdot C_{j}}}$$ (2)

The parameter β determined the combined noise in perceptual and decision processes, with infinite noise assuming a value of β = 0. For very large values of β, the probability of choosing the target with the lowest cost approaches 1. For intermediate values the probabilities are always ordered according to the cost (highest probability for lowest cost) but allow higher cost targets to be selected occasionally.

To assess the contribution of reward rate, target distance, and target size, we compared the full one-ahead model against the three submodels, obtained by removing each individual factor, using the Bayesian Information Criterion (BIC), with a lower BIC indicating a better fit [27,28].

The Bayesian Information Criterion (BIC) was used to compare models with different numbers of parameters:

BIC=2ln(L)+kln(N) (3)

where L is the likelihood of the data given the model, k is the number of degrees of freedom of the model and N is the total number of data points. The BIC allow models with different numbers of parameters to be compared, with the one with a lower BIC being preferable. The difference in the BIC scaled by 0.5 approximates the log of the Bayes factor, the likelihood that one model is better than another (27). A Bayes factor larger than 10 indicates strong evidence in favor of a model, and a value larger than 100 is considered decisive (28).

**Fig 3:**
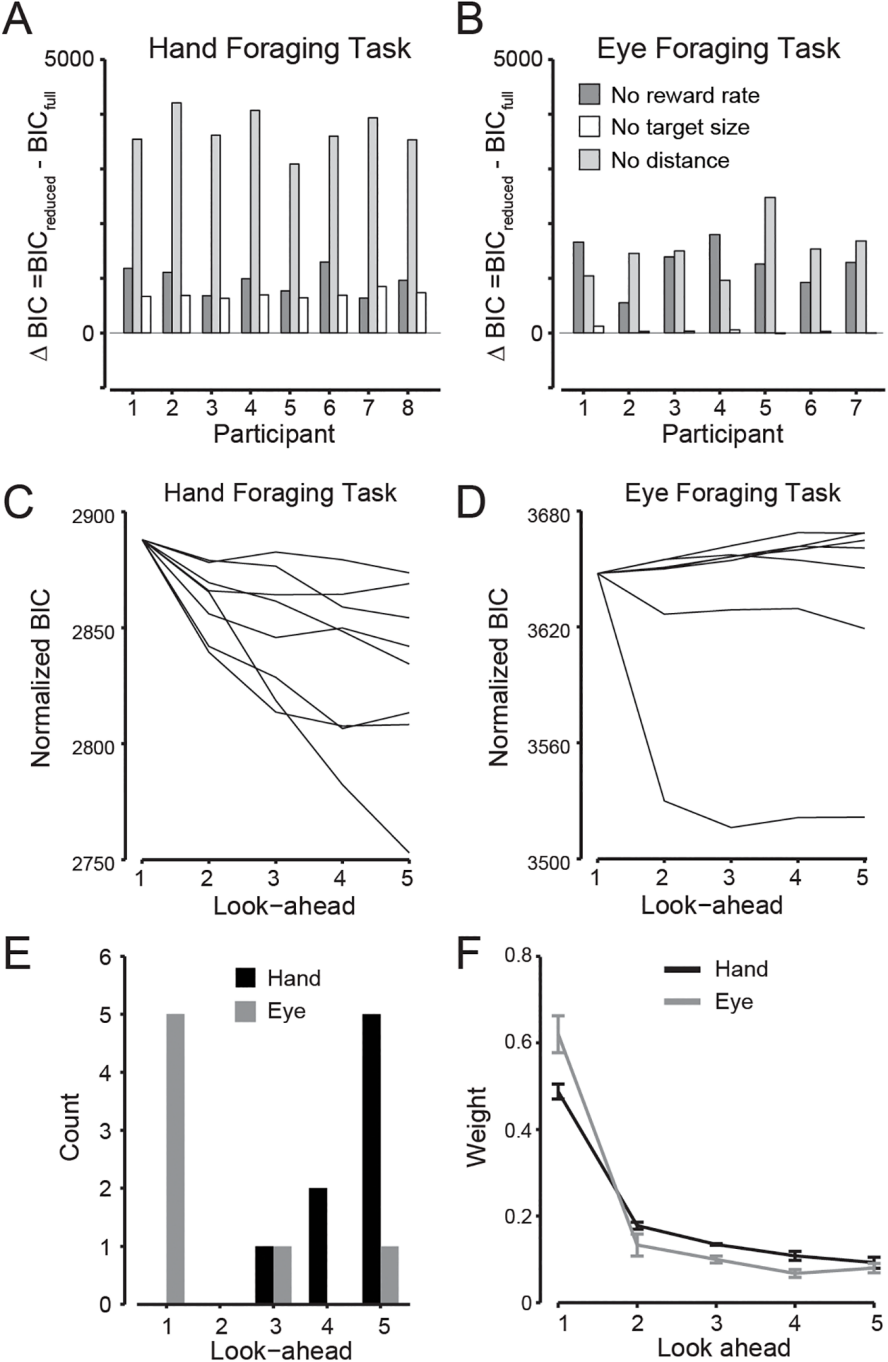
Model performance and look-aheads. **A-B**, Bayesian Information Criterion (BIC) for each participant in the hand and eye task used to compare models having different number of parameters, where smaller BIC scores are preferred. The difference in BIC shows the BIC relative to the full cost model for three different models in which one component of the cost is removed: no reward rate (dark grey bars), no target size (white bars), or no distance (medium grey bars). **C-D**, shows the BIC scores for each participant as a function of look-ahead for the hand and eye task, with BICs normalized to the mean score for the 1 look-ahead model. **E**, histogram showing the number of participants in the hand (black bars) and eye (grey bars) task whose best fitting model incorporated a given number of look-aheads. **F**, Average weights computed from participant means assigned to each look-ahead number in the model for the hand (black line) and eye (grey line) task taken from the 5 look-ahead model. Error bars represent ±1 SE.

Fig 3A and 3B show the change in BIC score (Δ BIC) going from the full model to each of the three submodels, for the hand and eye tasks respectively. The full model provided the best fit for all 8 participants in the hand task and for 4 of 7 participants in the eye task. In the other 3 participants who performed the eye task, the best model included reward rate and distance but not target size. However, this model and the full model had very similar BIC scores in all participants in the eye task (i.e., Δ BIC was very close to zero). For all participants in the hand task, the model omitting target distance was the poorest predictor of target choice by a large margin, and this model was also the poorest predictor in 5 of 7 participants in the eye task (with the model omitting reward rate being the poorest predictor in the other 2 participants). Overall, these results indicate that in both the hand and eye tasks, all three parameters of the full model—i.e., reward rate, target distance, and target size—influence choice behavior.

### Look ahead model

We next considered an extension of the model that could ‘look ahead’ to take into account potential future harvests when selecting the next harvest. When looking *n* harvests ahead (where *n* = 1 corresponds to the model already described), we consider each potential next harvest target *j* and all possible subsequent sequences of *n-*1 harvests (*k*, *l*, *m*, …). For each harvest we calculated the cost of each harvest with different weightings, λ, applied to future harvests. For example, when looking *n =* 5 steps ahead (i.e., *j*, *k*, *l*, *m*, *n*) the cost of choosing *j* as the next harvest is given by:

$$C_{j}=\underset{H(k,l,m,n)}{\min}(c_{ij}+\lambda_{1}c_{jk}+\lambda_{2}c_{kl}+\lambda_{3}c_{lm}+\lambda_{4}c_{mn})$$ (4)

where the minimum is taken over all quadruplets *H(k*, *l*, *m*, *n)* of potential harvested targets after the first harvest. Therefore *C*_*j*_ represents the smallest cost associated with making the next harvest target *j* when considering the next 4 targets. Again we used the softmax function to select the next harvested target. We modeled look aheads, *n*, from 1 to 5 targets (due the combinatorial nature of the problem it was not possible to consider look aheads of 6 or more). For a look ahead of *n*, the model had *n*+4 parameters. We also fit reduced models in which one of the three components in Eq 1 was set to zero.

Fig 3C and 3D show BIC scores as a function of the number of look-ahead harvests for each participant in the hand and eye tasks, respectively. Note that the curves have been vertically aligned so that they start from the same point, which is the mean BIC score, across participants, for the 1-look-ahead model. In the hand task, the BIC score tended to decrease (indicating a better fit) as a function of look-ahead steps whereas, in the eye task, the BIC score tended to increase for the majority of participants. The histogram in Fig 3E shows the number of participants best fit by models with 1 to 5 look-aheads. In the hand task, the best-fit model for all participants contained at least 3 look-aheads and, in 5 of the 8 participants, the 5-look-ahead model provided the best fit. With the exception of one participant, the BIC scores appeared to level off somewhat as the number of look-aheads increased. In the eye task, the 1-look-ahead model provided the best fit in 5 of 7 participants, with the other two participants’ choice behavior best fit by models the 3 and 5 look-aheads. Despite the variation across participants within each task group, there is a clear difference between the two tasks in the number of look-aheads. Whereas hand task all participants consider a sequence of forthcoming target choices when selecting the next target whereas, in the eye task, most participants chose targets on a harvest-by-harvest basis.

When considering the best-fit models for each participant, the average power exponent on the distance term (gamma in Eq 1) was 0.36 (SE = 0.09) for the eye task and 1.14 (SE = 0.23) for the hand task. Thus, the influence of target distance in determining target choice was close to linear in the hand task but compressive in the eye task, consistent with the idea that the additional cost of making larger eye movements was less than the additional cost of making larger hand movements.

In the model, different weights are assigned to the costs associated with each look-ahead step. To further assess the contribution of future harvests to the choice of target in the current harvest, we fit the 5-look-ahead full model to each participant. Fig 3F shows the average weights assigned to each look-ahead step in the hand and eye tasks. (Note that we normalize the weights for a given participant to sum to one.) For both tasks, the largest weight was assigned to the immediate choice option (i.e., 1-look-ahead), with this weight being higher in the eye task than the hand task, consistent with the finding that the 1-look-ahead model provided the best fit for most participants who performed the eye task. These effects were confirmed by a two-way look-ahead number by task ANOVA, which revealed a main effect of look-ahead number (F_4, 52_ = 130.0, p < 0.001) as well as a look-ahead by task interaction (F_4, 52_ = 4.6, p < 0.05).

**Fig 4:**
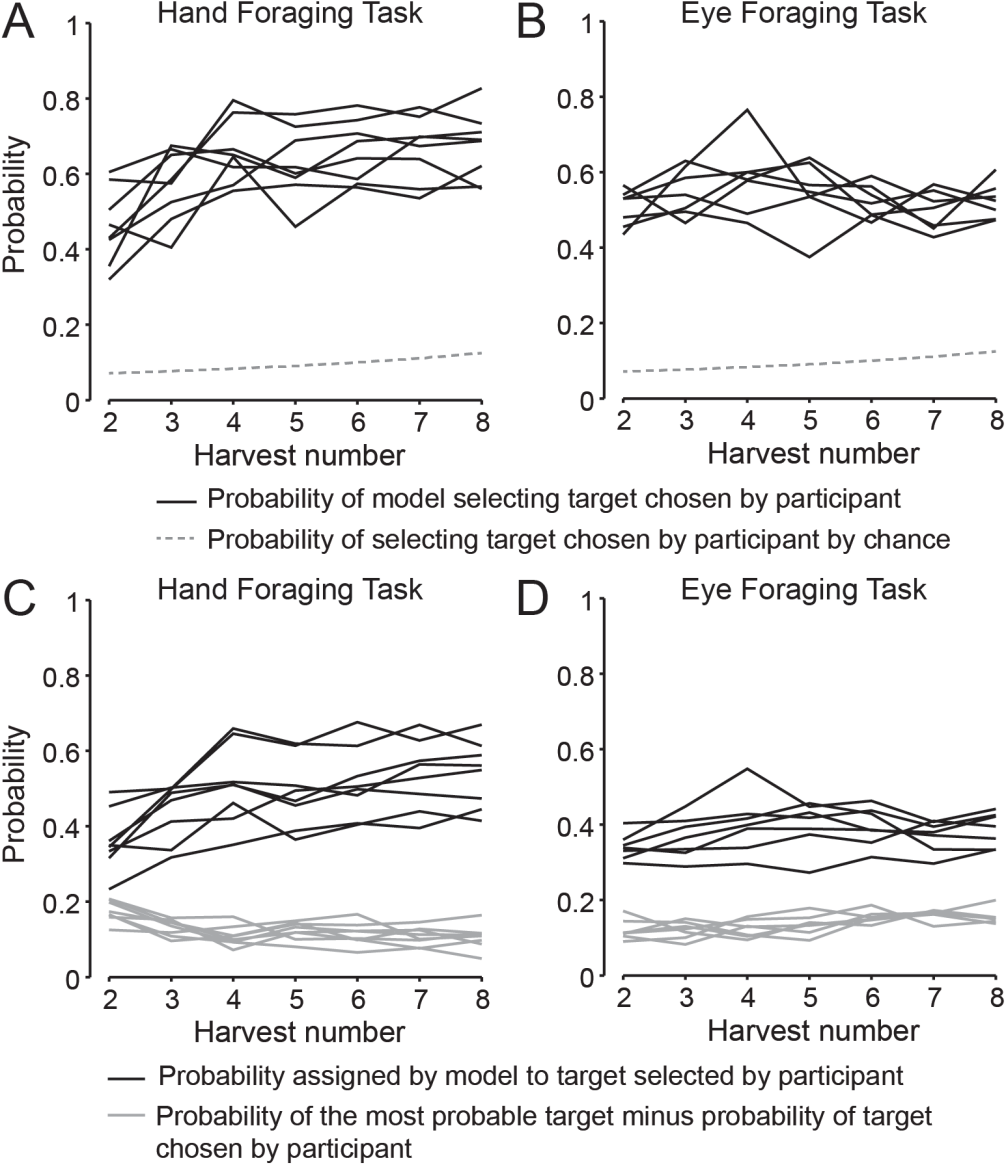
Target selection probabilities. **A-B**, The black lines show, for each participant in the hand and eye tasks, respectively, the average probability that the participant selected the target assigned the highest probability by the model for harvests 2–8. The dashed grey line show the probability that a target would be selected by chance, which increases slightly as targets are harvested. **C-D**, Black lines show the average probability, assigned by the model, to the target selected by each participant in the hand and eye tasks, respectively, for harvests 2–8. The grey lines show the probability of the most probable target minus the probability of target selected by the participant.

To evaluate the best-fit model for each participant (i.e., the full model with the number of look-aheads that provided the lowest BIC), we compared the actual target selections to those predicted by the model. At each harvesting step, the model assigns a probability of selection to each available (i.e., non-harvested) target. The black traces in Fig 4A and 4B show, for each participant in the hand and eye tasks respectively, the probability that the participant selected the target assigned the highest probability of selection by the model, as a function of harvest number. Overall, participants selected the highest probability target 61 percent of the time in the hand task and 53 percent of the time in the eye task. As is evident from the figure, across all harvests in both tasks, the probability of selecting the highest probability target was well above chance (dashed grey traces), which increases from 1/14 (0.071) to 1/8 (0.125) from the second to the eighth harvest.

The black traces in Fig 4C and 4D show the probability, assigned by the model, to the actual target selected by the participant, and the grey traces show the difference between the highest assigned probability and the probability assigned to the selected target. Although participants did not always select the target with the highest assigned probability, they generally selected a high probability target. Overall, the average ranking—from highest to lowest probability—of the selected target was 1.61 in the hand task and 1.96 in the eye task. These results are indicative of the fact that the probabilities assigned to the most probable few targets were often similar. Note that both the probability of selecting the highest probability target (black traces in Fig 4A and 4B) and the probability assigned to the selected target (black traces in Fig 4C and 4D) increased over the initial few harvests in the hand task, but were quite constant in the eye task. This observation is consistent with the fact that, in the eye task, the influence of target distance is relatively weak, which yields more target options with similar probabilities of selection.

To assess how well participants performed, we estimated each participant’s ‘optimal’ performance using an optimal planner that could look 5 harvests ahead at each harvest. Note that we could not use an optimal planner that considered all targets as this involved 15 factorial (~10^12) possible harvest orders, which is too many to evaluate. For each harvest choice, the optimal planner uses the predicted duration of each possible sequence of remaining harvests (up to 5) to select the best sequence in terms of maximizing rate of reward (given the remaining time). The predicted duration was determined separately for each participant based on that participant's estimated movement durations as a function of distance and target size (see above). This target-by-target choice was repeated until the trial time elapsed. We computed the efficiency of each participant's performance as the mean, over all trials from all conditions, of the ratio of the actual to 'optimal' points score. For the eye task, the average ratio was 0.95 and the ratio ranged from 0.85 to 0.98 across the participants. For the hand task, the average ratio was 0.91 and the ratio ranged from 0.84 to 0.96 across the participants. A t-test failed to show a significant difference between the two tasks (t_13_ = 1.63; p = 0.13). We also computed the ratio of the actual number of target harvested to the number harvested by the optimal planner. For the eye task, the average ratio was 0.95 and the ratio ranged from 0.85 to 0.99 across the participants. For the hand task, the average ratio was 0.92 and the ratio ranged from 0.84 to 0.99 across the participants. As with the points ratios, a t-tested failed to show a significant difference between the two tasks (t_13_ = 1.50; p = 0.16). Thus, in terms of both points and targets harvested, participants in both tasks were highly efficient and performed almost as well as the optimal 5-ahead planner.

### Features of selected targets over harvests

**Fig 5:**
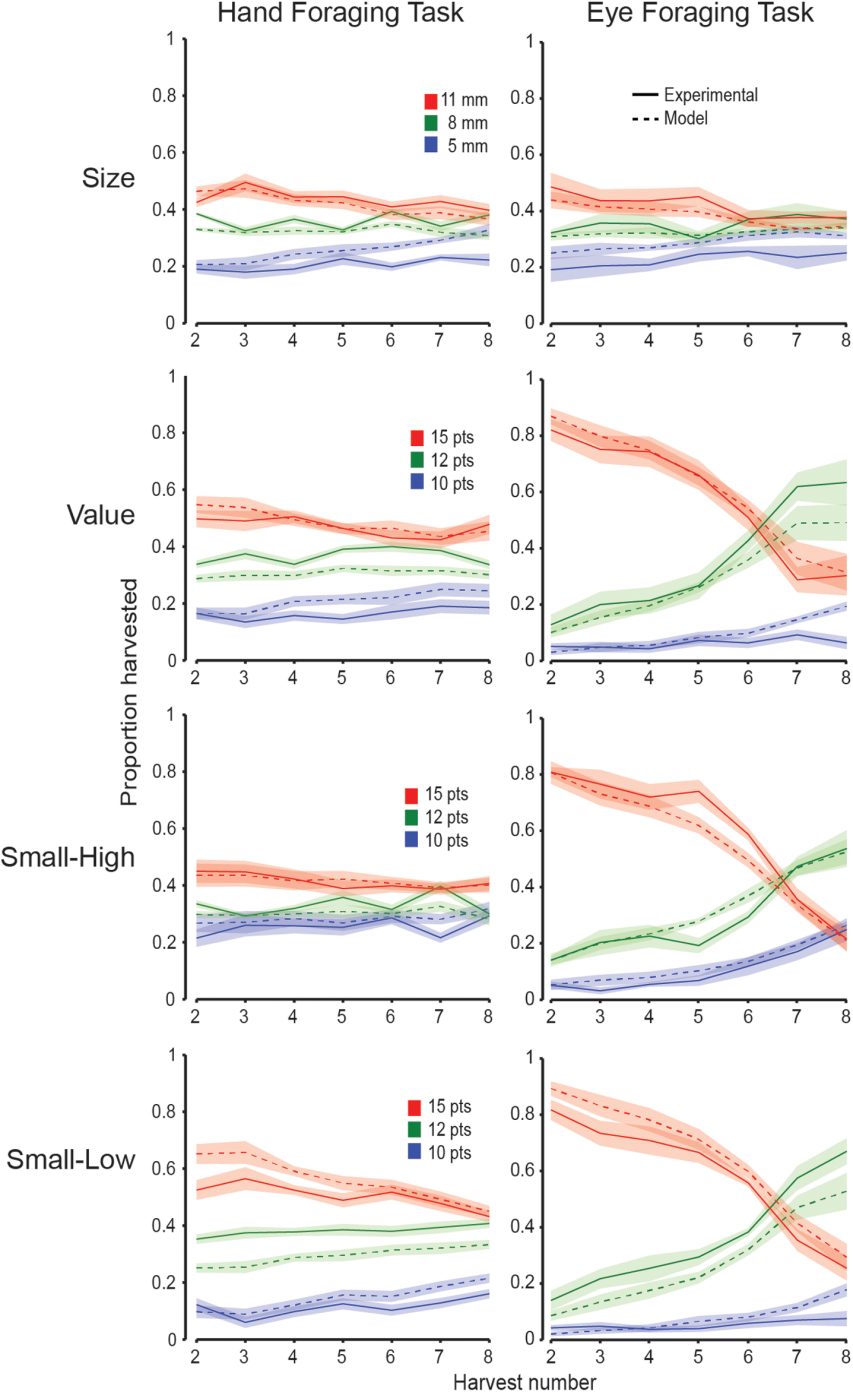
Target selection features. Proportion of targets of a given size or value selected from harvests 2–8. Proportions averaged across all participants in the hand (left column) and eye (right column) tasks. The red, green and blue traces show proportions for high, medium and low value harvests, respectively, or in the case of the size condition (top row), large, medium, and small target harvests, respectively. These proportions are shown for the experimental data (solid lines) as well as the model data (dashed lines). Shaded regions represent ±1 SE.

To examine how participants prioritized targets of varying value and size across harvests, for each participant and condition we calculated the proportion of targets, of a given size or value, that were selected on each successive harvest. Fig 5 shows these proportions, averaged across all participants, for harvests 2 through 8 in both the hand and eye tasks. The figure also shows the predicted proportions, averaged across participants, obtained with each participant’s best-fit model. (Note that the data from all four conditions the predicted together.) Qualitatively, it is evident that the model was able to capture the choice behavior of the participants in both tasks and in all four conditions.

In the eye task under conditions in which target value varied (i.e., the value, small-high, and small-low conditions), participants had a strong tendency to initially select high value targets right from the start (i.e., from harvest number 2 onwards), and largely ignored the size of the targets. When most or all of the high value targets were harvested, they then strongly favored the middle value targets. In contrast, under the corresponding conditions in the hand task, the influence of value on target selection was weaker, and varied considerably across conditions. Thus, although high value targets were always preferred, this preference was weaker when the high value targets were small (small-high condition) and stronger when the high value targets were large (small-low condition). A modest influence of target size was observed under the size condition in both tasks, even though size had little influence on choice behavior in the eye task in the other conditions. These results are consistent with the finding that movement related costs play a more significant role in shaping choice behavior in the hand task than the eye task. Participants in the hand task appeared to exploit the fact that they had enough time to harvest most, if not all, of the high value targets without having to harvest the high value targets first. Presumably, they were willing to harvest lower value targets in order to reduce movement related costs. In contrast, participants in the eye task tended to select the high value targets, presumably because they can do so without incurring large movement related costs.

### Movement distance

**Fig 6:**
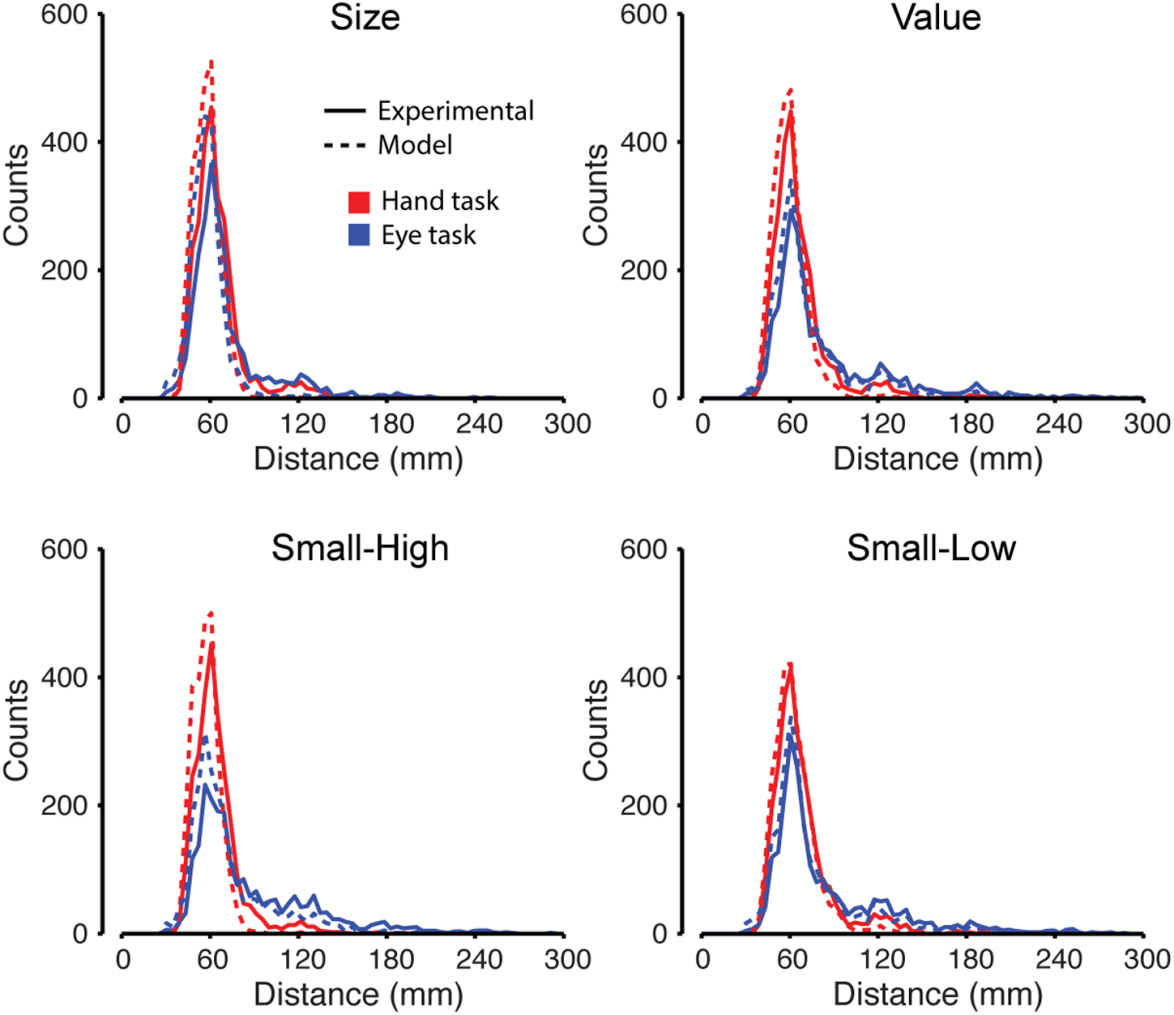
Distribution of movement distances. Frequency distributions of target-to-target distances for all actual (solid line) and predicted (dashed line) harvests in both the hand (red traces) and eye (blue traces) tasks for each condition averaged across participants. The pronounced peak at 60 mm reflects the separation distance of adjacent targets and the most common movement.

Fig 6 shows, for each of the conditions, frequency distributions of target-to-target distances for all actual and predicted harvests in both the hand and eye tasks averaged across participants. In both tasks, participants most often harvested targets that were directly adjacent (up, down, left, or right) to the previously harvested target and were therefore on average 60 mm away (corresponding to the mode of the distributions). Participants less frequently harvested adjacent but oblique targets, located on average 90 mm away, and targets located 2 or, even less frequently, 3 targets away. Although the distributions for the hand and eye tasks were similar in that adjacent targets were strongly preferred, a greater number of larger distances were seen in the eye task (blue traces) than the hand task (red traces). Two independent samples Kolmogorov-Smirnov tests revealed significant differences (p < 0.05) between the two tasks in the three conditions where target value varied (Fig 6), but not in the size condition where only target size varied (upper left subplot). Presumably, participants were more willing to move greater distances in the eye task, when value was manipulated, because of the lower movement-related costs involved. However, target size alone did not drive participants to move greater movement distances in the eye task.

### Constrained hand task

**Fig 7:**
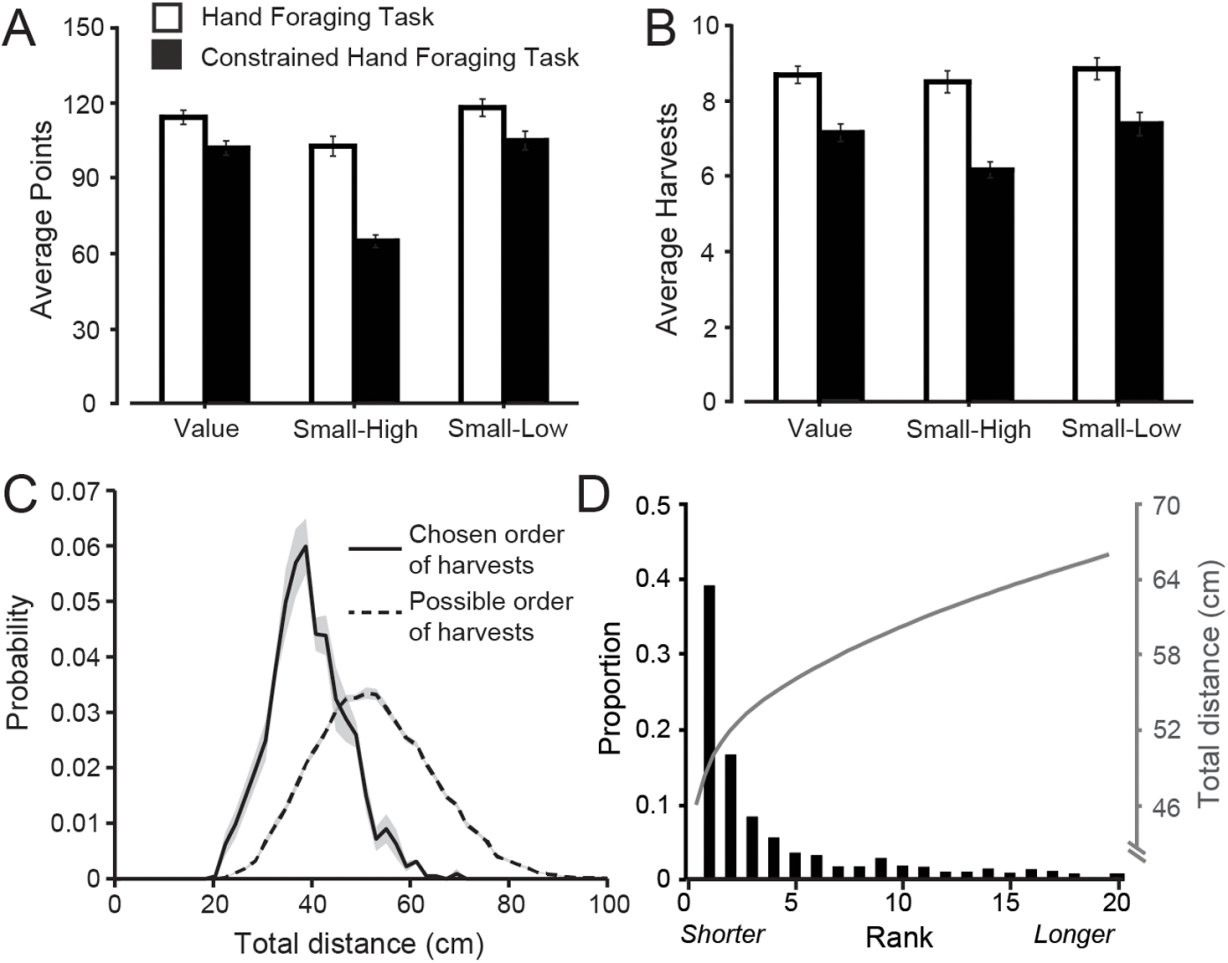
Performance in the constrained hand task. **A-B,** Average number of points and harvests, respectively, per trial across the three conditions examined in the constrained hand task. Error bars represent ±1 SE. **C**, Mean distributions, averaged across participants, of possible path distances (dashed line) and actual path distances (solid line) that participants chose. Shaded areas represent ±1 SE. **D**, The bars show the proportion of trials in which participants selected the shortest possible path (rank 1), the next shortest path (rank 2), and so on up to the 20^th^ shortest path. The grey line shows the total path length as a function of rank.

We included a constrained hand task, in which participants were required to harvest targets in order of decreasing value, for two reasons. First, comparing performance on the constrained and unconstrained (or ‘free’) hand tasks enables us to test whether sometimes selecting lower value targets before harvesting all high value targets (as in the unconstrained hand task), improves performance. Second, by focusing on the first 5 harvests (i.e., harvests of the high value targets) in the constrained task, we could assess how effectively participants minimize movement path distance. Fig 7A and 7B show the average number of points and harvests, respectively, per trial for both the free and constrained hand tasks in the three conditions examined in the constrained hand task. (Note that the size condition was not performed since the targets all had equal value.) On average, participants harvested all 5 high value targets and one or two mid-value targets in the constrained hand task. A task (2 levels: hand, eye) by condition (three levels: value, small-high, small-low) repeated measures ANOVA showed that participants harvested more points (F_1, 7_ = 68.0, p < 0.001) in the free choice task than the constrained task. A similar ANOVA showed that participants also harvested more targets (F_1, 7_ = 72.4, p < 0.001) in the free choice task than the constrained task. These findings indicate that participants’ inclination to sometimes forgo high value targets in the free task led to more optimal performance. The analysis also uncovered a task by condition interaction for both the number of harvested points (F_2, 14_ = 43.3, p < 0.001) and targets (F_2, 14_ = 6.2, p = 0.012). As shown in Fig 7A and 7B, for both variables, the largest discrepancy between tasks is seen in the small-high condition, where high value targets were small. In the constrained task, fewer targets were harvested when participants were required to harvest the small targets first. This could reflect a speed-accuracy trade-off, more challenging visual search, or both. However, even when the high value targets were large, the number of targets harvested in the constrained task was less than in the free choice task.

To assess how effectively participants minimized hand path distance, we computed, for each trial in the constrained task, the distance between successive targets for all 120 possible harvest orders of the first five targets. Fig 7C shows the distribution of possible path distances (dashed line) together with the distribution of actual path distances that participants chose. It is evident that participants selected harvest paths from the lower end of the distribution of all possible paths. The histogram in Fig 7D shows the proportion of trials in which participants selected the shortest possible path (rank 1), the next shortest path (rank 2), and so on up to the 20^th^ shortest path. (The gray solid line shows the average total distance of the ranked paths.) Participants chose the shortest path on close to 40% of trials and selected one of the 5 shortest paths on approximately 75% of trials. These data show that participants were efficient at rapidly harvesting a sequence of targets the resulted in a relatively short cumulative distance.

## Discussion

We examined sequential decision-making within the context of a rapid motor foraging task. Participants had a limited time to harvest targets of varying value, position, and size either by moving the hand to the targets (hand task) or fixating the targets (eye task). We fit a probabilistic ‘look ahead’ model in which target choice depended on a cost function that included rate of reward, target distance, and target size of up to the next 5 targets, with different weights allowed for the cost of each future target. We found that in the hand task, in comparison to the eye task, target choice was more strongly influenced by target distance and size, although target size did influence target choice in the eye task when target value was constant. In addition, participants in the hand task took into account a greater number of future targets, with most participants considering at least 5 future targets, compared to the eye task, with most participants only considering the next target. In a version of the hand task designed to examine route-finding efficiency, we found that participants were capable of selecting efficient routes through the targets that reduced total distance travelled. These results suggest that participants take into account the motor costs associated with different effectors so as to be efficient in foraging with both the eye and hand.

Studies examining the control of reaching movements have typically focused on movements to a single target, and contemporary models of reaching behavior have emphasized the trade-off between accuracy and effort [1–3,5,24]. A number of studies have recently examined the interplay between motor control and the decisions about where and when to reach during single movements. For example, it is has been shown that people factor into account their spatial movement variability to optimize performance when reaching towards target configurations with different reward and penalty regions [29–31] and similar optimization has been shown for temporal variability [19,32,33]. Recent work has investigated decision-making processes associated with selecting a single target from among multiple potential targets [15,for reviews, see 34–37]. To capture choice behavior in such tasks, additional factors need to be considered, including target value and biomechanical costs.

However, in many real world tasks people must make a series of choices, each involve multiple potential targets. In this situation, captured by the task we examined, potential costs associated with planning ahead need to be considered. In our task, we found that target value and distance influenced choice behavior in both the hand and eye tasks, with the relative influence of distance being stronger for the hand. Target distance is clearly related to biomechanical costs, especially in the hand task, but may also be linked to temporal costs associated with target search and selection. Although target size did not strongly affect movement time in either task, it nevertheless influenced choice in the hand task. Recently, it has been suggested that participants can improve their accuracy without incurring additional time by increasing the level of control [25]. In other words the trade-off between speed and accuracy can be altered through the cost of control. We suggest that participants’ preference for larger targets in the hand task may partially reflect the greater cost of control involved in attaining small targets. The trade-off between value and biomechanical costs in the hand task agrees with recent work by Cos and colleagues [38–40] showing that people can rapidly predict biomechanical costs associated with competing reaching actions [see also 41, 42].

Our probabilistic model suggests that participants in the hand task took into account a greater number of future harvests in comparison to participants in the eye task. Such ‘looking ahead’ in the hand task presumably enabled participants to trade off more effectively biomechanical and time costs with the value of the targets harvested. The ability to look ahead has been shown for a task in which participants reached through a series of fixed via-points which had to be visited in a prescribed order [43]. Our results also show that, in both the hand and eye tasks, participants placed the most weight on the current target option with each subsequent target option having progressively less weight in the decision. This finding can be linked to the phenomenon of ‘temporal discounting’ of reward which has been shown to influence the kinematics of single saccadic eye movements, with more rewarding targets leading to faster movements [44–48]. Our study shows that such temporal discounting acts across a sequence of future movements of both the eye and hand.

To our knowledge, our study is the first to quantitatively assess how costs and rewards associated with future targets influence current target choice during sequential target acquisition with *either* the hand or the eye. Thus, the results pertaining to each task, in isolation, are in themselves novel. A priori, it was not obvious that in the eye task participants would *only* consider the next target when selecting targets. By ‘looking ahead’ (i.e., considering multiple future targets, viewed in peripheral vision, while fixating the current target in order to harvest it), participants could have reduced, on average, the amplitude and therefore duration of each eye movement. It is also possible that they could have reduced the average distance between the remaining targets and the current fixation point, which may have facilitated search. On the other hand, it strikes us as impressive that, in the hand task, participants consider a number of future targets given the speeded nature of the task. That is, given the short harvesting window, it was not obvious, a priori, that participants would invest resources in looking ahead.

In terms of the comparison between the eye and hand tasks, we acknowledge that there are several differences between the tasks beyond the effector involved. Whereas target size directly determined the required accuracy of hand movements, this was not the case for eye movements. Given that the functional fovea is approximately 3° [49], it was simply not possible to implement similar accuracy constraints in the two tasks. We did not record eye movements in the hand task because it was difficult to obtain accurate recordings while participant generated very vigorous arm movements. However, based on previous work (as well as our observations), we can be quite certain that participants launched eye and hand movements towards each target in synchrony and maintained fixation at the target until the hand cursor arrived and the button was clicked [49–55]. Thus, the overall pattern of eye movements in the two tasks would have been similar. In both tasks, gaze shifts from one harvested target to the next and information about targets remaining to be harvested is provided by peripheral vision. Of course, the function of gaze differs between the two tasks. Whereas visual feedback is presumably used to help keep gaze on target in both tasks, in the hand task retinal and extraretinal signals are also used to guide the hand. Given the additional functions played by gaze in the hand task, it seems unlikely that gaze demands account for the reduced look-ahead behaviour observed in the eye task. However, we would emphasize that our goal was not to perfectly equate the two tasks, which are necessarily somewhat different. Rather, we wanted to keep each task as natural as possible, within the overall timing constraints, so that we could broadly compare the behaviours. We believe that the striking difference between the two tasks, in terms of look ahead behaviour, is likely quite robust and does not depend on subtle details of the two tasks.

Activity in sensorimotor areas of the brain has been shown to encode multiple potential reach targets prior to deciding between, and then reaching towards, one of these targets [56]. One interpretation of this activity is that it reflects competing movement plans prepared for multiple potential targets (Cisek and Kalaska, 2010), an idea consistent with recent behavioral studies showing spatial averaging when reaching towards multiple potential reach targets [57–62]. The formation of multiple motor plans may be an effective way of evaluating the costs associated with these alternatives [40]. In the current task, it is an open question whether participants may prepare competing immediate motor plans (i.e., from the current target to different potential targets), as well as plans for future actions.

Although numerous studies have examined how rewards and costs associated with individual targets are neurally represented [63–65], our results indicate that the brain must also represent, and evaluate, alternative routes involving multiple targets, potentially in parallel. A number of studies have identified brain regions that may play a role representing and planning such routes. Recent work on navigational planning in an open area has shown that, prior to goal-directed navigation, the rat hippocampus generates sequences of neural events encoding spatial trajectories from the current location to the known goal location [66]. These events may support a goal-directed, trajectory-finding mechanism that identifies important places and relevant behavioural paths and can be used to control future navigational behaviour. This, or a similar, mechanism may support the optimization of sequential reaching movements to objects is reachable space. In behavioural studies, route optimization has been studied using versions of the travelling salesperson problem in which participants attempt to select the shortest path when connecting a set of fixed targets [67]. This work, which has focused on the heuristics used to solve the problem, has shown that, when given ample time, humans are capable of very good performance levels, often with near optimal solutions [10–13]. Our results indicate that even under speeded conditions, participants are able to perform extremely well.

Although there are obviously many differences between natural foraging and the reaching task we examined, foraging theory [20,21] can nevertheless provide a framework for studying movement decisions in the context of competing costs and rewards [see 68]. In general, a key component of natural foraging involves deciding whether to engage with options as they are sequentially encountered. For example, work on patch foraging examines whether to remain in the current patch (i.e. to exploit) or switch to a new patch (i.e. to explore) which can incur a cost of time or effort [69,70]. In the most general setting there is both uncertainty as to the value of the current patch, which can vary with time as resources are depleted, and the value of other patches that could be visited. In such general settings it has been suggested that distinct neural processes appear to be engaged in choices within a patch and choices to move to forage a new patch [68,70], revealing aspects of the exploration-exploitation trade-off [71,72]. In our task, the main pressure is time within a patch and the assessment of target value, size and distance must occur rapidly leading to some uncertainty. Therefore our task examines the efficiency of foraging within a patch to extract valuable resources and the potential trade-off between looking far ahead (which can incur a time cost) and myopic decisions that can be faster but less efficient.

## Methods

### Participants

Eight women and seven men between 20 and 28 years of age participated in the present study after providing written, informed consent. All participants self-reported having normal or corrected-to-normal vision, being right handed, and being free of sensorimotor dysfunction. Participants were randomly assigned to one of two groups: the hand foraging group (n = 8) or the eye foraging group (n = 7). The experimental protocols were approved by the General Research Ethics Board at Queen’s University in compliance with the Canadian Tri-Council Policy on Ethical Conduct for Research Involving humans. Each experimental session lasted approximately one hour. Participants received $10 in compensation and an additional monetary sum based on the total points harvested during the experiment. Specifically, a 5¢ bonus was provided for every 250 points harvested, resulting in an additional payoff of between $4 and $5.

### Apparatus and stimuli

Seated participants viewed 15 circular targets located in 3 rows by 5 columns (see examples in Fig 1). To specify the locations of the targets on each trial, we began with a 3 x 5 grid with equal spacing of 6 cm in both dimensions, providing 15 initial grid locations. A set of 15 target locations was generated from these 15 grid locations by adding random vertical and horizontal offsets to each grid location, independently drawn from a uniform distribution over ± 11 mm. Thus, the positions of the targets, relative to the grid locations, changed from trial to trial. Targets in the display could be one of three sizes—5, 8, or 11 mm in radius—and have one of three point values—10, 12, or 15 points (Fig 1B). Participants also viewed a circular start position, 5 mm in diameter, located 6 cm closer to their body than the first row of targets and at the participant’s midline. All stimuli were presented using a visual display system, consisting of a CRT projector (Electrochrome 9500 Ultra) with a refresh rate of 120 Hz and a horizontal mirror through which participants viewed the images on a horizontal surface. Note that participants could not see their hand or arm.

In the hand foraging task, participants selected targets by moving a circular cursor (5 mm in diameter) linked to the position of a handle, grasped with the right hand, to each target. A successful harvest occurred when participants pressed a button fitted to the side of the handle with their index finger when the cursor overlapped with any portion of the target. The handle, which was attached to a lightweight manipulandum (Phantom 3.0, Sensable Technologies, Cambridge, MA), could freely rotate about its vertical axis and was mounted on an air sled allowing participants to move the handle by sliding over a horizontal surface. Optical encoders in the manipulandum measured the handle position at 1000 Hz and the state of the button was also recorded at 1000 Hz.

In the eye foraging task, participants selected targets by fixating their gaze on the targets. An infrared video-based eye-tracker (ETL 500 pupil/corneal tracking system, ISCAN, Burlington, MA, USA) was used to record gaze position of the left eye in the plane of the target display at 240 Hz. A bite bar was used to help stabilize the head. Gaze position was calibrated to the plane of the target display [for details, see 54] at the beginning of the experiment and recalibrated if drift in the recorded gaze signal occurred. The spatial resolution of gaze in the horizontal plane of the hand was 0.36° visual angle, corresponding to ~3 mm when gaze was directed to the center of the targets. In practice, gaze was recalibrated approximately once per participant, typically at around 75% of the duration of the experiment. The gaze signal was smoothed, on-line, using a running average filter computed over the last 50 samples (oversampled at 1000 Hz), which introduced a small time delay of 24.5 ms. In performing the gaze foraging task, participants almost always fixated one of the targets. That is, participants shifted their gaze directly from one target to the next. To determine which target was fixated, we simply took the closest target to the gaze position. A successful target selection was achieved when gaze was at a given target for 150 ms. Fixations less than 150 ms were rarely observed, as might be expected if participants (1) made corrective saccades after fixating a target they did not want to harvest or (2) briefly fixated targets to explore the scene. With the required fixation duration, the time between successive harvests was similar to the hand foraging tasks. Note that given that the size of the functional fovea, which is about 3° [49] or ~2.5 cm in the target plane in our task, it would not have been appropriate to require participants to align the recorded gaze position inside the targets in order to harvest them. Note that we did not record eye movements in the hand task because participants tended to make rapid and vigorous arm movements in this task which make obtaining stable gaze recordings difficult.

### Procedure

For a given trial, participants were instructed to harvest as many points as possible (which translated to a monetary bonus). At the start of each trial, the participant had to position the cursor, or fixate, the start position for a random time period of between 0.3 and 2.3 s. The target display then appeared, and the participant then had a fixed duration of 3.25 s to harvest targets. This duration was chosen so that on average subjects could average about half the targets on any given trial. At the moment a target was successfully harvested, the target turned grey and a brief tone (1000 Hz) was sounded for 50 ms. At the end of each trial, the total number of harvested points was presented on the target display. Participants completed one practice block of trials, consisting of a set of targets that were all of medium size (8 mm radius) and value (12 points). Participants then completed four experimental blocks of 50 trials, in counterbalanced order, with a 3–5 minute rest between blocks.

The four experimental blocks differed in how target size and value were combined (see Fig 1B). In the size condition, only target size was varied, with 5 targets of each size, while target value (12 points) was held constant. In the value condition, only target value was varied, with 5 targets of each value, while target size (8 mm) was held constant. For this condition, the low, medium, and high value targets were colored blue, green, and orange, respectively (in all other conditions, all of the targets were blue). In the small-high condition, the target size was negatively correlated with target value with the smallest targets being the most valuable. Participants were presented with 5 small targets of high value, 5 medium-sized targets of medium value, and 5 large targets of low value. Lastly, in the small-low condition, target size was positively correlated with value with the larger targets being the most valuable. Participants were presented with 5 small targets of low value, 5 medium-sized targets of medium value, and 5 large targets of high value. Before each block, participants were explicitly told the size-value pairing of the upcoming block. These blocks were completed in both the hand and eye foraging tasks.

Participants in the hand foraging group performed an additional constrained hand foraging task in which they were required to harvest targets in order of decreasing value. That is, participants were required to harvest all of the high value targets first, followed by the medium value targets, and then the low value targets. Participants could not harvest a target out of order; if a participant attempted to harvest a target out of sequence, no tone was sounded and the target did not change to gray. In this task, participants typically harvested between 6 and 7 targets and therefore we could assess their route-selection efficiency by comparing the actual route they selected through the first 5 (high value) targets to all possible 5-target routes (n = 120). In this task, participants performed three blocks of 50 trials, counterbalanced across participants, corresponding to the value, small-high, and small-low conditions described above.

### Analyses

For each harvest, we determined the duration from the previous harvest (i.e., duration between successive successful button presses) as well as the distance from the previous harvest, defined as the distance between successive target centers. In the hand foraging task, participants occasionally missed the target by pressing the button while the cursor was slightly off the target. Participants on average missed the target on 7% (SE = 0.01%) of harvest attempts in the free task and 8% (SE = 0.01%) in the constrained task. In the eye task, harvests were registered when gaze was closest to a given target for 150 ms and participants did not press a button. Thus, misses akin to those in the hand task did not occur. An alpha level of 0.05 was used for statistical tests and a Bonferroni correction was used for post hoc tests.

## Supporting information

S1 File Matlab mat file containing the data from all experiments. (MAT)

S2 File Matlab m file explaining the content and organization of the Matlab mat file. (M)
